# Symptom experience and subsequent mortality: results from the West of Scotland Twenty-07 study

**DOI:** 10.1186/1472-6963-6-158

**Published:** 2006-12-11

**Authors:** Alison M Elliott, Philip C Hannaford, Blair H Smith, Sally Wyke, Kate Hunt

**Affiliations:** 1 Department of General Practice and Primary Care, University of Aberdeen, Foresterhill Health Centre, Westburn Road, Aberdeen, AB25 2AY, Scotland, UK; 2 Department of Nursing and Midwifery, University of Stirling, Stirling, FK9 4lA, Scotland, UK; 3 MRC Social and Public Health Sciences Unit, Glasgow University, 4 Lilybank Gardens, Glasgow, G12 8RZ, Scotland, UK

## Abstract

**Background:**

Associations between symptom experience and mortality have rarely been investigated. One study has suggested that the number of symptoms people experience may be an important predictor of mortality. This novel and potentially important finding may have important implications but needs to be tested in other cohorts.

**Methods:**

858 people aged around 58 years were interviewed by nurses in 1990/1 as part of the West of Scotland Twenty-07 Study. They were asked about the presence of symptoms in the last month from a checklist of 33 symptoms. Measures of morbidity included symptom type (respiratory, musculoskeletal, gastrointestinal, mental health, neurological, systemic) and symptom summary measures looking at the number and impact of symptoms (total number; number participants tended to have; number participants did not tend to have; number which restricted usual activities; number which led to GP consultation). Hazard ratios for thirteen-year all-cause mortality were calculated for symptom types, symptom summary measures, and self-assessed health with and without adjustment.

**Results:**

On unadjusted analysis, and after adjusting for gender, socio-economic status and smoking, mortality was elevated in individuals reporting respiratory, systemic and mental health symptoms. After additional adjustment for chronic conditions and self-assessed health, only the association between mental health symptoms and mortality remained significant. On unadjusted analysis, and after adjusting for gender, socio-economic status and smoking, mortality was elevated in individuals with many (≥ 6) symptoms in four of the symptom summary measures examined. These relationships were no longer significant after additional adjustment for chronic conditions and self-assessed health. A clear trend of increasing mortality as self-assessed health became poorer was observed. This pattern remained statistically significant after adjustment for gender, socio-economic status, smoking, chronic conditions and the total number of symptoms experienced.

**Conclusion:**

Symptoms often thought of as minor may have important consequences later in life especially for those reporting mental health-related symptoms or those experiencing many symptoms. In this study however, self-assessed health appeared to be a better predictor of mortality than the type or number of symptoms experienced, even when the tendency to have and impact of the symptoms were taken into account.

## Background

There has been growing interest in recent years in researching symptoms in the community [1,2], particularly since symptoms are drivers of health care utilisation and have been shown to be the principal reason for clinic visits in about half of all outpatient encounters [2]. Previous population-based studies of a range of symptoms [3-10] have provided useful information on the prevalence of symptoms within different socio-demographic groups, examined symptom burden and looked at the impact of symptoms in terms of quality of life, reduced functioning and use of health services. While some of these studies have examined the relationship between symptoms and the presence of chronic disease or self-assessed health, none of them have investigated the relationship with mortality.

Only one previous study has investigated the relationship between symptom burden and mortality [11]. Sha et al. found that individuals with higher symptom scores were significantly more likely to die over the subsequent year than individuals with no or few symptoms, even after adjustment for age, sex, race, chronic conditions, self-assessed health, depression and anxiety. This suggests that the number of symptoms people experience may be an important predictor of mortality. This novel finding, if replicated, may have important implications. Further evidence is needed however to see whether these findings exist in other studies. In addition, since a simple count of the number of symptoms experienced by individuals is a crude measure with limitations, further work is needed to investigate whether this relationship is stronger for more detailed symptom measures.

In order to explore this we examined, in a UK cohort study, the associations between type and number of symptoms and mortality. We were particularly interested in investigating the relationship between the burden of symptoms with different impact and mortality. We hypothesised that summary measures of symptom experience, which take into account the number of symptoms experienced, any tendency to have the symptoms and any impact on functioning, would be better predictors of mortality than simple counts of symptoms. In addition, since self-assessed health has been found to be a strong predictor of morbidity and mortality in previous studies [12,13], we also examined the relationship between self-assessed health and mortality to see if the relationship is mediated through symptoms.

## Methods

### Participants and procedures

The data were from the Regional sample of the West of Scotland Twenty-07 study, a longitudinal study of health amongst three cohorts, aged 15, 35 and 55 when first studied in 1987/88 (see elsewhere for full details) [14-16]. In brief, in order to get a representative sample of people in each age group a two stage sampling strategy was used. The sampling frame was the 1986 Voluntary Population Survey, an enhanced electoral register with details of names, sex and ages of all household members maintained by Strathclyde Regional Council (SRC). For ethical reasons SRC required written consent from potential participants to forward their names to the MRC for possible inclusion in the study. The University of Glasgow Ethics Committee for non-clinical research approved the West of Scotland Twenty-07 study.

Here we use data on the 55 year old cohort as there are few deaths to date in the younger cohort. For the 55 year old cohort 2155 people were approached by SRC and 1196 (55%) gave written consent to have their name transferred. Of these, 1042 (87%) agreed to participate in the study when approached. The cohort was first followed up in 1991 when 858 (82%) of the original 1042 men and women were interviewed at around 58 years of age. This contact included more detail on symptoms (such as whether or not activities were limited by the symptom) than the baseline survey.

### Measures

During a nurse-led interview conducted in participants' homes, individuals were asked whether they had experienced any of 33 listed symptoms (including respiratory/ENT, musculo-skeletal, gastrointestinal, mental health, neurological, systemic (general) and other symptoms: Table 1). These symptoms were presented to participants on cards listing up to six symptoms at a time. Participants were asked whether they had experienced each symptom in the last month and whether it was a symptom they tended to have (i.e. one that they experienced often). For each symptom experienced in the last month participants were also asked whether it had caused them to cut down on their usual activities, and whether they had consulted a general practitioner for this occurrence of that symptom. Self-reports of longstanding chronic conditions were elicited using an extended version of the question used in the UK General Household Survey [17,18]. Each condition was coded to a condition group (International Classification of Diseases chapter heading) according to the British Royal College of General Practitioners coding scheme [19]. Self-assessed health in the previous year was measured using the question: "In the last year how would you rate your general health – excellent, good, fair or poor?" A wide range of personal and social information was also collected including gender; occupational social class (non-manual, manual); housing tenure (owned/mortgaged, rented from council/housing association, privately rented and other); car ownership (no, yes); Carstairs deprivation category (1–2 (least deprived), 3–5, 6–7 (most deprived))[20]; and smoking status (never, ex, current).

### Death notifications

Participants in the West of Scotland Twenty-07 Study were 'flagged' at the National Health Service central registries in Scotland and England at the start of the study in 1987/88. At regular intervals the study is notified of the date and cause of any deaths occurring in the cohort.

### Data analysis

The data were analysed using SPSS for Windows. Deaths occurring in the cohort up to the end of May 2004 were included in the analysis and used to calculate hazard ratios for thirteen-year all cause mortality. In order to identify which factors were unadjustedly associated with mortality, unadjusted hazard ratios and their 95% confidence intervals were calculated for: gender; occupational social class; housing tenure; car ownership; deprivation category; smoking status; presence of a musculoskeletal condition; presence of a respiratory condition; presence of a digestive condition; presence of a cardiovascular condition; presence of a mental health condition; the total number of chronic conditions suffered and self-assessed health.

In order to examine the association between type of symptom (respiratory/ENT, musculoskeletal, gastrointestinal, mental health, neurological, and systemic) and mortality, unadjusted hazard ratios and their 95% confidence intervals were calculated for each symptom type, using absence of such symptoms as the referent group in each case. Cox regression was then used to adjust these hazard ratios for potential confounding from gender, socio-economic status, smoking, chronic conditions, and self-assessed health. We did not adjust for all of our measures of socio-economic status, since these measures were highly correlated. We used housing tenure only as the measure of socio-economic status in our adjustments.

In order to examine the association between number of symptoms and mortality, symptoms were categorised into five symptom summary measures: total number of symptoms experienced in the last month; number of symptoms experienced in the last month which participants tended to have; number of symptoms experienced in the last month which participants did not tend to have; number of symptoms experienced in the last month which resulted in cutting down on usual activities; and number of symptoms experienced in the last month which resulted in consulting a general practitioner. Within each summary measure, symptoms were aggregated so that individuals were categorised as having at baseline (1991) 0, 1, 2, 3–5, or ≥ 6 such symptoms (this division was made to ensure we had adequate numbers of individuals in each category). The exception to this approach occurred for the symptom summary measure indicating the number of symptoms experienced in the last month which participants did not tend to have, where individuals were aggregated as having at baseline 0, 1, 2, ≥ 3 such symptoms because of the small numbers of people with higher symptom counts in this summary measure. Unadjusted hazard ratios and their 95% confidence intervals were calculated for different levels within each symptom summary measure, using absence of such symptoms as the referent group in each case. Cox regression was then used to adjust these hazard ratios for potential confounding.

In order to examine the association between self-assessed health and mortality, unadjusted hazard ratios were calculated for poor, fair and good health, with excellent health as the referent group. Hazard ratios were then adjusted for potential confounding from gender, socio-economic status, smoking, chronic conditions, and total number of symptoms in the last month.

## Results

A total of 170 deaths (19.8 percent of the cohort) occurred in the cohort in the thirteen years between 1991 and 2004. Most deaths were caused by cardiovascular disease (n = 62) or cancer (n = 56). Women were significantly less likely to have died than men, and car owners were significantly less likely to have died than those not owning a car (Table 2). Individuals in manual occupations, those who rented their house from the council or a housing association, those in higher deprivation categories and smokers were all significantly more likely to have died than those in the referent group for each variable.

Individuals with cardiovascular and respiratory conditions were more likely to have died than people without these conditions (Table 3). The presence of musculoskeletal, digestive or mental health conditions did not appear to affect mortality. There was a strong association between number of reported chronic conditions and mortality. A clear trend was seen between self-assessed health and mortality, with mortality increasing as self-assessed health became poorer (Table 3).

The most commonly reported symptoms in the last month were stiff or painful joints (38.8%), difficulty sleeping (29.7%), headaches (25.8%), back trouble (23.1%) and always feeling tired (21.9%). For further details on symptom prevalence see elsewhere [8]. Table 4 shows the hazard ratios for thirteen-year all cause mortality by type of symptom. On unadjusted analysis participants with respiratory/ENT, mental health or systemic symptoms had a significantly higher mortality risk than those not reporting such symptoms. These results remained statistically significant after adjustment for socio-demographic factors. However, after additional adjustment for the presence of a respiratory or cardiovascular condition and self-assessed health, only mental health symptoms showed a significant association with mortality (HR, 95%CI: 1.42, 1.02 to 1.97).

**Figure 1:**
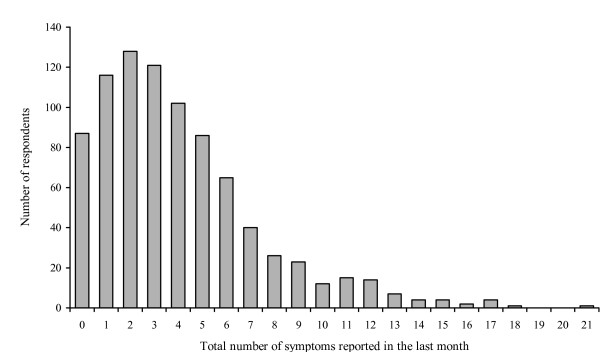
Distribution of total number of symptoms experienced in the last month based on self report from a list of 33 symptoms.

The total number of symptoms experienced in the last month reported by respondents at baseline ranged from 0 to 21, with a median of two symptoms (Figure 1). Table 5 shows the hazard ratios for thirteen-year all cause mortality by number of symptoms for each of the five symptom summary measures examined. Four of the symptom summary measures (total number of symptoms; number of symptoms which participants tended to have; number of symptoms which restricted usual activities; and number of symptoms which resulted in consulting a general practitioner) showed very similar results. As the number of symptoms reported rose, the chances of dying tended to increase, although there was no clear trend in any of the summary measures with increasing number of symptoms. Individuals who reported having six or more symptoms in each of these four summary measures were significantly more likely to have died than those without such symptoms. These results remained statistically significant after adjustment for gender, socio-economic status and smoking. After additional adjustment for the presence of respiratory and cardiovascular conditions the hazard ratios were smaller and the results were of only borderline significance. After further adjustment for self-assessed health no significant associations remained. There was no relationship between number of symptoms and mortality in the fifth symptom summary measure (number of symptoms experienced in the last month which participants did not tend to have).

Self-assessed health was strongly associated with mortality. Respondents with self-assessed poor health had a mortality risk over seven times (HR, 95% CI: 7.72, 3.62 to 16.44) that of participants reporting excellent health status. This association remained significant even after adjustment for gender, socio-economic status, smoking, chronic conditions and the total number of symptoms in the last month (adjusted HR, 95%CI for poor vs excellent self-assessed health: 4.46, 1.95–10.21).

## Discussion

### Summary of main findings

On unadjusted analysis, and after adjusting for gender, socio-economic status and smoking, mortality was elevated in individuals reporting respiratory, systemic and mental health symptoms suggesting that some types of symptoms have a greater impact on mortality than others. After additional adjustment for a history of respiratory and cardiovascular conditions and self-assessed health, only the association between mental health symptoms and mortality remained statistically significant. This finding suggests that the presence of mental health symptoms has important implications for subsequent health. On unadjusted analysis, and after adjusting for gender, socio-economic status and smoking, mortality was elevated in individuals reporting many (≥ 6) symptoms for four of the five symptom summary measures examined, suggesting that the presence of many symptoms (of different frequency and severity) in late mid-life predicts subsequent mortality. The absence of any association among individuals reporting symptoms in the last month that they did not tend to have highlights the importance of considering the tendency to have and/or impact of symptoms rather than simple symptom counts. After additional adjustment for chronic conditions and self-assessed health in the last year none of the associations between symptom groups and mortality remained statistically significant.

### Comparison with existing literature

Few studies have examined the relationship between type of symptoms and mortality in community populations, tending, when done, to focus on respiratory and depressive symptoms. A significant relationship between respiratory symptoms (such as breathlessness, wheeze and cough) and all-cause mortality has consistently been found in previous population studies, even after adjustment for confounding factors [21-25]. Our findings are broadly in line with these previous studies, although our association with mortality was lost after adjustment for the presence of respiratory and cardiovascular conditions and self-assessed health. Less consistent results have been found in population studies assessing the relationship between mental health symptoms and mortality. Vogt et al. [26] reported no relationship. Everson-Rose et al. [27] found a significant association after adjusting for age, gender and race. However, after additional adjustments for socio-economic status, lifestyle behaviours, functional limitations and chronic health conditions the relationship was eliminated. Wulsin et al. [28] found that all-cause mortality was directly associated with depressive symptoms, with higher mortality risks as depressive symptom scores increased. Our findings support Wulsin et al. and highlight that mental health symptoms may be important independent predictors of subsequent mortality in late mid-life.

Only one previous study has investigated the reporting of symptom burden in relation to mortality [11]. As part of a multi-centre study in the USA looking at 12 physical symptoms in 3498 adults aged 60 and over, Sha et al. found that individuals with higher symptom scores were significantly more likely to have died over the subsequent year than individuals with no or few symptoms. These results remained significant even after adjustment for age, sex, race, chronic conditions, self-assessed health, depression and anxiety. Our findings are broadly in line with Sha et al's, showing a two- to three- fold increase in mortality with increasing number of total symptoms reported when examined unadjustedly or after adjustment for socio-economic factors. Unlike Sha et al, however, our associations were smaller and no longer statistically significant after adjustment for self-assessed health. We examined a different, smaller population and different symptoms over a longer time period. Sha et al examined a smaller range of (physical only) symptoms, used simpler numeric summaries and did not include any measure of their impact. On the other hand, some of the symptoms they examined might be considered more serious (e.g. chest pain) than those examined in our study and this may explain why Sha et al. found a stronger relationship between symptoms and mortality than we did. In addition, they studied an older cohort of individuals (mean age at baseline 69 compared to 58 years in our study). Self-reported symptoms can arise from a variety of causes, including the early stages of disease which has yet to be detected, self-limiting minor illnesses which have no important long-term consequences, or from a known long-standing condition. The underlying mix of causes is likely to differ substantially with age; with older age groups more likely to have a higher percentage of symptoms related to chronic, serious or undetected conditions.

In our study, self-assessed health was strongly associated with mortality. This finding is in line with previous research, which has reported a two to seven fold increase in mortality among those with poor compared with excellent self-assessed health, after adjusting for clinical and medical history measures [12,13]. Our findings suggest that self-assessed health in the last year may be a better predictor of future mortality than the number of symptoms experienced, even when the tendency to have and impact of these symptoms are taken into account. When assessing their own health individuals are likely to take into account a range of factors such as medical health status, functional ability, physical fitness, experience of symptoms, psycho-social well-being, lifestyle and health behaviours [29,30]. A measure of symptom burden may be a sub-component of self-assessed health and therefore less able to predict mortality.

### Strengths and limitations of this study

Strengths of this study were its population-based design, the large number and range of symptoms covered and the detailed social information collected. The study was able to examine underlying morbidity in terms of self-reported symptoms, chronic conditions and self-assessed health. Although we had a relatively small number of participants, we had a long period of follow-up, enabling us to look at subsequent mortality over a 13-year period. This was a longer follow-up than in previous research [11] and resulted in a larger number of deaths available for analysis, thereby increasing statistical power (although there were insufficient data to permit cause-specific mortality analyses). An important limitation was the fact that although we asked about a large number of symptoms (over 30), most were largely minor, self-limiting in nature. Different relationships may have been found if we had included more serious symptoms (e.g. chest pain, weight loss etc). In addition, the analyses were based on self-reported data, which were not cross-checked with medical records. The experience of symptoms however, is by definition subjective and many of the symptoms (and some of the longstanding chronic conditions) will not have been presented to general practice, hampering attempts to validate the self-reported symptoms against medical records. Participants in the analyses were all aged around 58 years at the time of interview. As already highlighted it is likely that different age groups will report different numbers of symptoms and chronic conditions. It is also likely that their perceived self-assessed health will be different, as indicated by previous studies [31-33]. Whether symptom experience is related to mortality in other age groups, and whether self-assessed health would remain a stronger predictor of mortality than symptoms needs to be tested in other studies. Finally, our measures of symptom experience and self-assessed health had different time reference points (last month and last year respectively). It is noteworthy that self-assessed health over the last year appeared to be a stronger predictor of mortality than symptoms experienced in the previous month.

## Conclusion

This study has shown that symptoms often thought of as 'minor' or self-limiting may have important consequences later in life, especially for those reporting mental-health related symptoms or those experiencing many symptoms. Further work is needed to ascertain how best to help health-care professionals differentiate between symptoms which have serious outcomes and those which do not. Our observation that a single question about self-assessed health is a better predictor of mortality than various combinations of symptom experience points towards more research into understanding the utility of this measure.

## Competing interests

The author(s) declare that they have no competing interests.

## Authors' contributions

AME was involved in the conception and design of this piece of work, performed the statistical analysis, was involved in the interpretation of the work and drafted the manuscript. PCH was involved in the conception and design of this piece of work, interpreting the data and revising the manuscript critically. BHS was involved in the conception and design of this piece of work, interpreting the data and revising the manuscript critically. SW was involved in the conception and design of this piece of work, interpreting the data and revising the manuscript critically. KH was involved in the original study from which the data were drawn, the conception and design of this piece of work, interpreting the data, helping to draft parts of the manuscript and revising the manuscript critically. All authors read and approved the final manuscript.

## Pre-publication history

The pre-publication history for this paper can be accessed here:



## Figures and Tables

**Table 1:** List of 33 symptoms inquired about as part of the West of Scotland Twenty-07 study*

**Respiratory/ENT symptoms**
Colds or flu
Persistent cough
Palpitations or breathlessness
Sinus, catarrh or blocked nose
Sore throat
Wheezy chest
**Musculoskeletal symptoms**
Sore or stiff back
Stiff or painful joints
Muscle tightness or tension
**Gastro-intestinal symptoms**
Constipation
Stomach pain or cramps
Diarrhoea
Indigestion
Poor appetite
Sickness or nausea
Piles or haemorrhoids
**Mental health symptoms**
Difficulty sleeping
Nerves or tension
Difficulty concentrating
Worrying over every little thing
Feeling depressed
**Neurological symptoms**
Headaches
Trembling hands
Fainting or dizziness
**Systemic (general) symptoms**
Feeling generally run down
Always feeling tired
Hot and sweating a lot
Cold sweats
**Other symptoms**
Trouble with ears
Trouble with eyes
Skin rash or skin problems
Trouble with teeth or mouth
Kidney or bladder problems

*Symptoms were not presented to participants in this order or in these groupings

**Table 2:** Hazard ratios for thirteen-year all cause mortality by gender, socio-economic factors and smoking

**Socio-demographic factors**	**n in sample**	**Died n (%)**	**Unadjusted Hazard Ratio (95% CI)**
**Gender**			
Male	399	97 (24.3)	1.00
Female	459	73 (15.9)	0.63 (0.47 to 0.86)
			
**Social class**			
Non manual	361	51 (14.1)	1.00
Manual	497	119 (23.9)	1.80 (1.29 to 2.49)
			
**Housing tenure**			
Owned/mortgaged	474	62 (13.1)	1.00
Rented from council/housing association	352	100 (28.4)	2.35 (1.71 to 3.23)
Private rented and other	31	7 (22.6)	1.90 (0.87 to 4.15)
			
**Car ownership**			
No	319	88 (27.6)	1.00
Yes	538	81 (15.1)	0.51 (0.38 to 0.69)
			
**Deprivation category**			
1+2	135	17 (12.6)	1.00
3–5	407	72 (17.7)	1.43 (0.84 to 2.42)
6+7	314	81 (25.8)	2.17 (1.29 to 3.66)
			
**Smoking status**			
Never	285	29 (10.2)	1.00
Ex	239	42 (17.6)	1.80 (1.12 to 2.89)
Current	334	99 (29.6)	3.24 (2.14 to 4.90)

**Table 3:** Hazard ratios for thirteen-year all cause mortality by chronic conditions and self-assessed health

**Chronic conditions**	**n in sample**	**Died n (%)**	**Unadjusted Hazard Ratio (95% CI)**
**Presence of musculoskeletal condition**			
No	439	90 (20.5)	1.00
Yes	419	80 (19.1)	0.91 (0.67 to 1.23)
			
**Presence of respiratory condition**			
No	705	128 (18.2)	1.00
Yes	153	42 (27.5)	1.59 (1.12 to 2.25)
			
**Presence of digestive condition**			
No	683	129 (18.9)	1.00
Yes	175	41 (23.4)	1.32 (0.93 to 1.87)
			
**Presence of cardiovascular condition**			
No	627	95 (15.2)	1.00
Yes	231	75 (32.5)	2.45 (1.81 to 3.33)
			
**Presence of mental health condition**			
No	730	138 (18.9)	1.00
Yes	128	32 (25.0)	1.37 (0.93 to 2.01)
			
**Number of chronic conditions**			
0	107	11 (10.3)	1.00
1	211	23 (10.9)	1.07 (0.52 to 2.20)
2	222	48 (21.6)	2.18 (1.13 to 4.20)
3	147	33 (22.4)	2.33 (1.18 to 4.61)
≥ 4	171	55 (32.2)	3.69 (1.93 to 7.05)
			
**Self-assessed health**			
Excellent	118	8 (6.8)	1.00
Good	398	53 (13.3)	2.01 (0.95 to 4.22)
Fair	238	66 (27.7)	4.55 (2.18 to 9.47)
Poor	103	42 (40.8)	7.72 (3.62 to 16.44)

**Table 4:** Hazard ratios for thirteen-year all cause mortality by type of symptom

**Type of symptom**	n in sample*	**Died n (%)**	**Unadjusted Hazard Ratio (95% CI)**	**Hazard ratio (95% CI) adjusted for socio-demographic factors**^**1**^	**Hazard ratio (95% CI) adjusted for socio-demographic factors and chronic conditions**^**2**^	**Hazard ratio (95% CI) adjusted for socio-demographic factors, chronic conditions and self-assessed health**^**3**^
**Respiratory/ENT symptoms**						
Does not have symptoms	424	66 (15.6)	1.00	1.00	1.00	1.00
Has symptoms	434	104 (24.0)	1.60 (1.17 to 2.17)	1.44 (1.06 to 1.97)	1.24 (0.89 to 1.71)	1.10 (0.79 to 1.54)
						
**Musculoskeletal symptoms**						
Does not have symptoms	418	78 (18.7)	1.00	1.00	1.00	1.00
Has symptoms	440	92 (20.9)	1.12 (0.83 to 1.51)	1.14 (0.84 to 1.55)	1.15 (0.84 to 1.56)	0.99 (0.72 to 1.35)
						
**Gastro-intestinal symptoms**						
Does not have symptoms	552	101 (18.3)	1.00	1.00	1.00	1.00
Has symptoms	306	69 (22.5)	1.29 (0.95 to 1.76)	1.31 (0.96 to 1.78)	1.30 (0.95 to 1.77)	1.13 (0.82 to 1.56)
						
**Mental health symptoms**						
Does not have symptoms	461	70 (15.2)	1.00	1.00	1.00	1.00
Has symptoms	397	100 (25.2)	1.78 (1.31 to 2.41)	1.79 (1.31 to 2.45)	1.66 (1.21 to 2.28)	1.42 (1.02 to 1.97)
						
**Neurological symptoms**						
Does not have symptoms	581	112 (19.3)	1.00	1.00	1.00	1.00
Has symptoms	277	58 (20.9)	1.11 (0.81 to 1.53)	1.08 (0.78 to 1.49)	0.99 (0.71 to 1.37)	0.81 (0.58 to 1.13)
						
**Systemic symptoms**						
Does not have symptoms	553	92 (16.6)	1.00	1.00	1.00	1.00
Has symptoms	305	78 (25.6)	1.63 (1.21 to 2.21)	1.50 (1.10 to 2.05)	1.28 (0.93 to 1.76)	1.00 (0.71 to 1.41)

* Numbers in each of the five symptom categories add to 858 the total number in the cohort. Numbers given are relevant for unadjusted hazard ratios. Numbers in the sample for each of the adjusted analyses varies only slightly since there was little missing data for the factors we have adjusted for (as can be seen from tables 1 and 2).

^1 ^Adjusted for gender, housing tenure and smoking status

^2 ^Adjusted for gender, housing tenure, smoking status and the presence of a respiratory or cardiovascular condition

^3 ^Adjusted for gender, housing tenure, smoking status, the presence of a respiratory or cardiovascular condition and the number of symptoms present in the last month

**Table 5:** Hazard ratios for thirteen-year all cause mortality by number of symptoms in five symptom categories

		n in sample*	**Died n (%)**	**Unadjusted Hazard Ratio (95% CI)**	**Hazard ratio (95% CI) adjusted for socio-demographic factors**^**1**^	**Hazard ratio (95% CI) adjusted for socio-demographic factors and chronic conditions**^**2**^	**Hazard ratio (95% CI) adjusted for socio-demographic factors, chronic conditions and self-assessed health**^**3**^
**Number of symptoms in the last month**	**0**	87	9 (10.3)	1.00	1.00	1.00	1.00
	**1**	116	16 (13.8)	1.32 (0.58 to 2.99)	1.40 (0.62 to 3.17)	1.21 (0.53 to 2.75)	1.24 (0.54 to 2.82)
	**2**	128	27 (21.1)	2.13 (1.00 to 4.54)	2.06 (0.96 to 4.41)	1.94 (0.90 to 4.15)	1.99 (0.93 to 4.29)
	**3–5**	309	60 (19.4)	1.99 (0.99 to 4.00)	1.99 (0.98 to 4.03)	1.74 (0.85 to 3.55)	1.40 (0.68 to 2.86)
	**≥ 6**	218	58 (26.6)	2.76 (1.37 to 5.58)	2.53 (1.24 to 5.16)	2.03 (0.99 to 4.19)	1.38 (0.65 to 2.89)
							
**Number of symptoms in the last month which participants tended to have**	**0**	120	15 (12.5)	1.00	1.00	1.00	1.00
	**1**	141	18 (12.8)	0.99 (0.50 to 1.97)	1.06 (0.53 to 2.10)	1.00 (0.50 to 1.99)	0.99 (0.50 to 1.97)
	**2**	138	28 (20.3)	1.68 (0.90 to 3.15)	1.66 (0.88 to 3.13)	1.62 (0.86 to 3.05)	1.51 (0.80 to 2.86)
	**3–5**	291	59 (20.3)	1.70 (0.96 to 2.99)	1.60 (0.90 to 2.80)	1.44 (0.81 to 2.56)	1.14 (0.64 to 2.06)
	**≥ 6**	168	50 (29.8)	2.56 (1.44 to 4.57)	2.23 (1.24 to 4.02)	1.85 (1.01 to 3.37)	1.15 (0.61 to 2.18)
							
**Number of symptoms in the last month which participants did not tend to have**	**0**	588	123 (20.9)	1.00	1.00	1.00	1.00
	**1**	156	29 (18.6)	0.85 (0.57 to 1.28)	0.90 (0.60 to 1.36)	0.93 (0.61 to 1.40)	0.98 (0.65 to 1.49)
	**2**	59	11 (18.6)	0.89 (0.48 to 1.64)	1.18 (0.63 to 2.20)	1.20 (0.64 to 2.25)	1.16 (0.62 to 2.18)
	**≥ 3**	55	7 (12.7)	0.61 (0.28 to 1.30)	0.71 (0.33 to 1.53)	0.67 (0.31 to 1.44)	0.72 (0.33 to 1.55)
							
**Number of symptoms in the last month which resulted in cutting down on usual activities**	**0**	526	84 (16.0)	1.00	1.00	1.00	1.00
	**1**	130	30 (23.1)	1.50 (0.99 to 2.28)	1.45 (0.95 to 2.21)	1.40 (0.92 to 2.14)	1.19 (0.77 to 1.82)
	**2**	66	18 (27.3)	1.74 (1.03 to 2.93)	1.54 (0.90 to 2.61)	1.41 (0.82 to 2.41)	1.05 (0.60 to 1.83)
	**3–5**	97	22 (22.7)	1.54 (0.96 to 2.46)	1.35 (0.84 to 2.18)	1.17 (0.72 to 1.90)	0.85 (0.51 to 1.40)
	**≥ 6**	39	16 (41.0)	3.21 (1.88 to 5.49)	2.42 (1.39 to 4.20)	1.72 (0.97 to 3.06)	1.21 (0.67 to 2.21)
							
**Number of symptoms in the last month which resulted in consulting the GP**	**0**	457	72 (15.8)	1.00	1.00	1.00	1.00
	**1**	166	39 (23.5)	1.55 (1.05 to 2.29)	1.49 (1.00 to 2.21)	1.48 (0.99 to 2.20)	1.38 (0.93 to 2.06)
	**2**	91	19 (20.9)	1.39 (0.84 to 2.31)	1.46 (0.88 to 2.43)	1.36 (0.82 to 2.27)	1.13 (0.67 to 1.91)
	**3–5**	96	24 (25.0)	1.63 (1.02 to 2.61)	1.51 (0.94 to 2.43)	1.34 (0.83 to 2.16)	1.08 (0.66 to 1.75)
	**≥ 6**	48	16 (33.3)	2.51 (1.46 to 4.31)	1.96 (1.13 to 3.41)	1.58 (0.91 to 2.77)	1.13 (0.63 to 2.01)

* Numbers in each of the five symptom categories add to 858 the total number in the cohort. Numbers given are relevant for unadjusted hazard ratios. Numbers in the sample for each of the adjusted analyses varies only slightly since there was little missing data for the factors we have adjusted for (as can be seen from tables 1 and 2).

^1 ^Adjusted for gender, housing tenure and smoking status

^2 ^Adjusted for gender, housing tenure, smoking status and the presence of a respiratory or cardiovascular condition

^3 ^Adjusted for gender, housing tenure, smoking status, the presence of a respiratory or cardiovascular condition and self-assessed health
